# Omit needless words: Sentence length perception

**DOI:** 10.1371/journal.pone.0282146

**Published:** 2023-02-24

**Authors:** Nestor Matthews, Folly Folivi

**Affiliations:** Department of Psychology, Denison University, Granville, OH, United States of America; Federal University of Paraiba, BRAZIL

## Abstract

Short sentences improve readability. Short sentences also promote social justice through accessibility and inclusiveness. Despite this, much remains unknown about sentence length perception—an important factor in producing readable writing. Accordingly, we conducted a psychophysical study using procedures from Signal Detection Theory to examine sentence length perception in naive adults. Participants viewed real-world full-page text samples and judged whether a bolded target sentence contained more or fewer than 17 words. The experiment yielded four findings. First, naïve adults perceived sentence length in real-world text samples quickly (median = 300–400 ms) and precisely (median = ~90% correct). Second, flipping real-world text samples upside-down generated no reaction-time cost and nearly no loss in the precision of sentence length perception. This differs from the large inversion effects that characterize other highly practiced, real-world perceptual tasks involving canonically oriented stimuli, most notably face perception and reading. Third, participants significantly underestimated the length of mirror-reversed sentences—but not upside-down, nor standard sentences. This finding parallels participants’ familiarity with commonly occurring left-justified right-ragged text, and suggests a novel demonstration of left-lateralized anchoring in scene syntax. Fourth, error patterns demonstrated that participants achieved their high speed, high precision sentence-length judgments by heuristically counting text lines, not by explicitly counting words. This suggests practical advice for writing instructors to offer students. When copy editing, students can quickly and precisely identify their long sentences via a line-counting heuristic, e.g., “a 17-word sentence spans about 1.5 text lines”. Students can subsequently improve a long sentence’s readability and inclusiveness by omitting needless words.

## Introduction

Omit needless words. That self-exemplifying advice from a writing style guide [1] helps generate the clear and succinct writing that science writers value. Science writers can measure the clarity and succinctness of their writing via readability indices. Many readability indices depend—inversely—on two variables: word length and sentence length [2–7]. Unfortunately, word length can remain beyond the science writer’s control when the relevant science requires multi-syllable words. Fortunately, science writers can control their sentence length, and some readability research has identified sentence length as the best single measure of grammatical complexity [8]. Shortening sentences—by omitting needless words—improves readability [2, 3, 8–10].

Shortening sentences to improve readability also promotes social justice. Evidence for this comes from research ethics boards requiring informed consent forms to have readability at or below the 8^th^ grade level. Doing so fosters a demographically fair distribution of research costs and research benefits. This embraces the justice principle described in ethics documents such as the Belmont Report [11], and the World Medical Association’s Declaration of Helsinki [12]. Along these lines, the United States government advanced socially inclusive writing on October 13, 2010 by passing the Plain Writing Act [13]. The act subsequently inspired International Plain Language Day celebrated annually on October 13^th^ by the International Plain Language Association. The association recommends keeping average sentence length between 15 and 20 words and limiting individual sentences to no more than 35 words [14]. In sum, these diverse organizations have converged on a central point: briefer sentences for broader audiences.

Writing briefer sentences for broader audiences not only promotes social justice, it can also saves lives. Evidence for this comes from professional health organizations, whose public communication guidelines emphasize limits on sentence length. For example, the U.S. National Institutes of Health’s guidelines for written health information recommend limiting sentence length to 20 words or fewer [15]. Even more cautiously, the U.S. Centers for Disease Control recommends that sentences not exceed 10 words [16]. Restricting sentences to 10 rather than 20 words allows some wiggle room for medically necessary multi-syllable words. This follows from the fact that many readability formulas permit swapping word length for sentence length to maintain a desired reading grade level [2–7]. The American Medical Association and the U.S. National Institutes of Health recommend 6^th^-8^th^ grade readability for public health information [15–19]. Such recommendations have inspired a growing body of research that explores the readability of patient information for diverse medical matters. Examples include the readability of patient information on dementia [20], mammography for breast cancer screening [21], obstetrics and gynecology [22], andrology [23], orthopedics [24], podiatry [25], hip arthroscopy [26], and ophthalmology [27].

Still other readability studies have taken a step further, demonstrating associations between short sentences and improved reading comprehension. Examples include linking short sentences to improved comprehension of informed consent forms [28], patient education materials [29], and clinical trials [30]. This link matters because readability—a property of the text—merely sets the stage for reading comprehension, which entails complex reader-and-text interactions. Indeed, although reading comprehension is an end goal, writers can only directly control their own text’s readability—mostly through sensitivity to their sentence length.

The present study investigated how adults perceive sentence length, and had both applied and basic research motivations. The applied research motivation stemmed from the first author’s 21 years of experience evaluating undergraduate science writing, and desire to produce more readable science writers. Science writers often hinder the readability of their own writing by using long sentences. Does this reflect a perceptual failure, i.e., a limitation in precisely perceiving sentence length? To answer this question, we tested predictions from three pre-registered hypotheses about sentence length perception, each rooted in a distinct basic visual phenomenon. These basic visual phenomena include (1) numerosity sensitivity, (2) perceptual learning, and (3) scene syntax.

### Numerosity sensitivity hypothesis

Numerosity sensitivity refers to how precisely one perceives the number of elements in a set. In the present study, numerosity sensitivity corresponds to how precisely one perceives the number of words or text lines in a sentence. The numerosity sensitivity hypothesis parsimoniously posits that sentence length perception depends only on mechanisms already used to quantify other stimuli in the environment. Such mechanisms presumably evolved because the capacity to precisely register the number of predators, prey, or conspecifics conferred survival and reproductive advantages.

Numerosity researchers typically distinguish two numerosity mechanisms. One mechanism—subitizing—provides fast, confident, and error-free number judgments for small set sizes, typically one to four items [31–33]. The other mechanism—the approximate number system (ANS)—provides comparatively slower, less confident, and less precise numerosity estimates that generally follow Weber’s Law [34–40]. In principle, participants could use either or both of these numerosity mechanisms to judge sentence length. For example, the ANS could reasonably estimate the number of words in sentences that exceed the subitizing range, i.e., contain more than four words. Alternatively, or in addition, participants could use a “groupitizing” strategy [33, 41]. This entails perceptually organizing a sentence’s words into a small number of text lines, then subitizing those to estimate the sentence’s word-count by proxy.

The numerosity hypothesis makes predictions that arise from behavioral and physiological findings. Behavioral experiments show that participants directly sense numerosity *per se*, rather than deriving numerosities from related stimulus attributes like area, density, or texture [42–46]. Likewise, physiological experiments in monkeys [47–51], young human children [52, 53], and human adults [36, 54–60] have identified intraparietal sulcus (IPS) activity that tracks numerosities *per se*. Critically, numerosity-specific activity in the IPS occurs regardless of whether the task requires judging the number of visual stimuli or auditory stimuli [49]. This level of stimulus independence would render numerosity-based sentence-length judgements robust to orientational variability in visually presented text. Therefore, the numerosity hypothesis predicts that the precision of sentence length judgments will not depend on text orientation. For the same reason, the numerosity hypothesis further predicts that text orientation will not affect participants’ biases toward underestimating or overestimating sentence length.

### Perceptual learning hypothesis

The perceptual learning hypothesis posits that sentence length perception depends on the readers’ familiarity and expertise with words written in standard orientation. This orientation-dependence connects the present study to inversion effects—performance impairments caused by flipping stimuli to non-standard orientations. Inversion effects already emerged in psychological research by 1899 [61], perhaps owing to their salience. Additionally, inversion effects generalize to diverse stimuli and tasks. Examples include the perception of faces [62–66], body parts [67], mammograms [68], artificial objects (“greebles”) [69, 70], oriented shapes [71], change detection [72, 73], lexical decisions [74, 75], word identification [76], and reading [77]. Importantly for the perceptual learning hypothesis, inversion effects tend to increase with one’s level of perceptual expertise [68, 69]. This demonstrates that learning plays a role in generating inversion effects. Stated another way, the ability to extract visual information can depend on orientation specific practice [65, 66, 68]. Given these findings, the perceptual learning hypothesis predicts more precise sentence length judgments for standard text than for flipped text.

A second prediction from the perceptual learning hypothesis arises from an electroencephalograph (EEG) experiment on recognizing standard versus inverted faces. Compared to standard faces, inverted faces generated distinct EEG signals and "noisier" facial recognition performance, evidenced by increases in false positives and false negatives alike [64]. Accordingly, the perceptual learning hypothesis predicts that flipped text will generate increases in false positives and false negatives alike. In the present experiment, false positives and false negatives correspond to, respectively, overestimating and underestimating a target sentence’s length relative to a fixed length.

Requiring participants to judge a target sentence’s length relative to a fixed length facilitates analyzing lapses, i.e., non-sensory errors. Non-sensory errors can arise from various sources, including inattention, motivation failures, or motor errors. In principle, unfamiliarity with flipped text could reduce participants’ motivation on flipped-text trials. To the extent this occurs, flipped text would more frequently generate random guessing, i.e., lapsing, regardless of target-sentence length. Incorrect responses to target sentences that differ dramatically in length from the fixed (comparison) sentence length provide strong evidence for lapses. Analyzing error patterns across a wide range of sentence lengths therefore allows distinguishing genuine sensitivity reductions (errors near the comparison sentence length) from lapses. Either or both of these will increase when flipping the text—according to the perceptual learning hypothesis. The perceptual learning hypothesis also predicts that increased guessing on flipped-text trials will not alter participants’ biases toward underestimating versus overestimating sentence length.

### Scene syntax hypothesis

Scene syntax refers to the fact that, in real-world scenes, particular targets occur in some locations more often than in others [78–80]. The same holds for written English. For example, page numbers typically appear in the margins. Section headings typically appear above their sections. Figure captions appear near their figures. Left-justified right-ragged text appears more often than right-justified left-ragged text. In other words, non-random probabilities characterize the spatial organization—the scene syntax—of written English. These prior probabilities—whether in real-world scenes or in English text—contribute to a spatio-temporal priority map for allocating attention [81–85]. The map fosters briefer visual searches for targets occurring at higher priority (higher probability) locations and times [78–80].

The scene syntax hypothesis predicts that vertically or horizontally flipping the text would generate a systematic bias toward underestimating sentence length. This directional prediction arises from the prior probabilities of written English, which one reads from left-to-right and top-to-bottom. A typical multi-line English sentence will reach the right edge of the page, then wrap around to the next line’s left edge. Flipping the text reverses a multi-line sentence’s wrap-around pattern, moving text into locations that would never otherwise occur in a typically written English sentence. More specifically, in multi-line sentences, flipping the text moves words from higher to lower priority map positions [81–85]. This increases the probability of missing some of the flipped sentence’s words: “If you don’t find it often, you often don’t find it” [86]. The missed words result in underestimating flipped sentence length. Note that a bias toward underestimating sentence length would not necessarily alter the precision of the sentence length judgments. In other words, the scene syntax hypothesis predicts that flipping the text will bias participants’ sentence-length judgments toward underestimation without altering their precision.

### Cognitive strategy and the “mischievous sentence”

Beyond the predictions from the hypotheses described above, another prediction arose from our desire to understand the cognitive strategy participants use when judging sentence length. Our participants’ task required judging whether the target sentence on each trial had more or fewer than 17 words. During the experiment’s instruction phase, we informed participants that a 17-word sentence typically spans ~1.5 text lines. That information accurately described four of our five 16-word sentences. However, our stimulus set also contained a 16-word “mischievous sentence”. The mischievous sentence began near the right edge of the page, completed the next line, then concluded near the left edge of its third line. Therefore, the mischievous sentence nominally spanned three lines, unlike any of the other 16-word sentences which nominally spanned two lines. If participants judged sentence length by explicitly counting words, comparable error rates would occur on the 16-word mischievous sentence and the other 16-word sentences. By contrast, heuristically counting text lines would generate significantly more errors on the (three-line) 16-word mischievous sentence than on the other (two-line) 16-word sentences. In short, the mischievous sentence served as a probe to evaluate the cognitive strategy participants used when judging sentence length.

### Hypotheses summary & predictions

To summarize, the three pre-registered hypotheses tested here make the following predictions about the precision and bias in sentence length perception.

1. The numerosity sensitivity hypothesis predicts (a) equal precision for flipped and standard text, and (b) non-biased responding.
2. The perceptual learning hypothesis predicts (a) worse precision for flipped than for standard text, and (b) non-biased responding.
3. The scene syntax hypothesis predicts (a) equal precision for flipped and standard text, and (b) a bias toward underestimating sentence length.

Additionally, judging sentence length by counting text lines—rather than individual words—predicts worse performance on our 16-word “mischievous sentence” than on other 16-word sentences.

## Methods

### Ethics, preregistration, and reproducibility

On September 23, 2021, Denison University’s Institutional Review Board approved the experiment reported here. The experiment adheres to the October 2008 Declaration of Helsinki [12]. To minimize HARKing and P-Hacking [87, 88], we pre-registered the experiment’s hypotheses, methods, and statistical analysis plan with the Open Science Framework on October 11, 2021 [https://osf.io/3k5cn]. On November 4, 2021, we collected data with the written informed consent of each participant. To promote reproducibility, the Open Science Framework [https://osf.io/89myj/] contains the complete data set and all software needed to replicate the experiment and the statistical analyses. In the Results, we distinguish pre-registered from exploratory analyses [89].

### Participants

The *Prolific* online crowdsourcing service recruited 88 adults who had identified English as their first language before learning about the present experiment. All 88 participants completed the experiment online.

### Materials & apparatus

We initially generated python code for the experiment using the “Builder” interface in PsychoPy 2021.2.3 [90]. The “Builder” automatically converted the PsychoPy code to PsychoJS, and then pushed that javascript to the *Pavlovia* online platform. We provided our *Prolific* participants with a web link to access the experiment’s javascript hosted on *Pavlovia*.

In response to *Prolific’s* prompt about permissible devices—“Which devices can participants use to take your study?”—we selected only the “desktop” option. Therefore, we presume that participants used desktop computers when completing the experiment online.

### Online timing precision

A 2020 study evaluated two aspects of online timing precision for PsycoPy/PsychoJS: reaction time precision, and visual stimulus duration variability [91]. PsychoPy/PsychoJS reached online reaction time precision under 4 ms using most browser/OS combinations, and sub-millisecond precision using Chrome for both Windows and Linux. Similarly, PsychoPy/PsychoJS reached inter-trial stimulus duration variability of less than 5 ms across most browser/OS combinations. The actual stimulus durations undershot and overshot the desired stimulus durations about equally often.

### Sentence stimuli

To promote applicability to real-world settings, we created stimuli that mimic what writers typically see when writing or proof-reading their own text. Specifically, we took Microsoft Word versions of actual manuscripts published recently in *PLOS ONE* [92, 93], bolded one sentence per page, then screen-captured the entire page. We repeated this until obtaining five unique samples at each of 15 bolded-sentence-lengths that ranged from 10 to 24 words. This generated (5 * 15 =) 75 unique writing samples with a standard text-orientation. We flipped those 75 standard-orientation samples around the vertical axis to create mirror-reversed stimuli, and around the horizontal axis to create upside-down stimuli.

**Fig 1:**
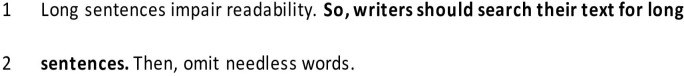
Conceptual visualization of standard text. Participants judged whether the target sentence (bolded) on each trial contained more or fewer than 17 words.

**Fig 2:**
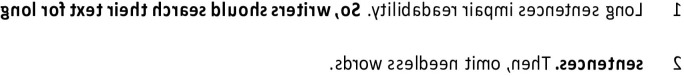
Conceptual visualization of mirror-reversed text. Participants judged whether the target sentence (bolded) on each trial contained more or fewer than 17 words.

**Fig 3:**
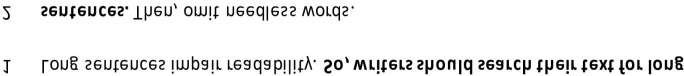
Conceptual visualization of upside-down text. Participants judged whether the target sentence (bolded) on each trial contained more or fewer than 17 words.

On each trial, participants viewed a page of text presented for two seconds. Each page contained a bolded target sentence embedded among numerous non-bolded distractor sentences. Randomly across trials the text had either a standard or a flipped orientation; mirror-reversed for one group, upside-down for another group. As a conceptual visualization, Figs 1–3 respectively show a standard, upside-down, and mirror-reversed 9-word target sentence embedded in two lines of text. The supporting information contains full-page illustrations of a 17-word target sentence, shown at each text-orientation: standard, mirror-reversed, upside-down (S1–S3 Figs). The 17-word target sentence spans ~1.5 lines of text. The supporting information also contains our “mischievous sentence”, which has only 16 words yet spans three lines of text (S4–S6 Figs).

### Task & feedback

Participants pressed either the left or right arrow key to signal whether the bolded sentence contained, respectively, fewer or more than 17 words. Immediate feedback followed each response. Specifically, the monitor displayed for one second either the word “correct” in lowercase green letters or the word “WRONG” in uppercase red letters.

### Procedure

The instructions informed participants about the stimuli and task, and that bolded target sentences would contain fewer versus more than 17 words equally often. Importantly, the instructions also provided participants with the heuristic that a 17-word bolded sentence would typically span ~1.5 lines of text. After receiving computerized instructions, participants proceeded through demonstration trials, practice trials, and trials for analysis.

### Demonstration trials

Participants familiarized themselves with the stimuli across 10 demonstration trials. Each required passively viewing a sample text page containing a 17-word bolded target sentence embedded among non-bolded distractor sentences. The first five demonstration trials exemplified standard text and the next five exemplified flipped text. On flipped-text trials, the computer displayed mirror-reversed text to half the participants, and upside-down text to the other participants.

### Practice trials

Practice trials comprised 2-second presentations of a standard or flipped text page containing either 10 or 24 words—the two extremes of our sentence-length range. To reduce random responding from our online participants we implemented an attention-and-comprehension check, which the *Prolific* platform encourages. This check required each participant to meet criterion accuracy during the practice trials. Specifically, after the 20th practice trial, the computer evaluated whether the participant performed significantly better (binomial probability p<0.001) than chance. Participants who met criterion accuracy after 20 practice trials proceeded immediately to the next phase: trials for analysis. The other participants continued practicing until reaching criterion accuracy. If the participant failed to meet criterion accuracy after 60 practice trials, the experiment ended and the software directed the participant to the debriefing.

### Trials for analysis

Each participant completed 140 trials for analysis, with standard and flipped text randomly interleaved across trials. The 70 trials within each of those two text-orientation conditions comprised 5 unique text-page stimuli at each of 14 bolded-target sentence lengths. These sentence lengths ranged from 10 to 24 words, excluding the 17-word bolded-target stimuli at the center of our sentence length range.

As an incentive, participants who met criterion accuracy on practice trials and completed all 140 trials for analysis received the greater of the following two rewards.

1. $7 for performing the trials for analysis at only 50% correct or less, or
2. 10 cents for each correct trial-for-analysis response, i.e., between $7.10 and $14.

Overall, the experiment typically required about 20 minutes.

### Research design

We administered the independent variables via a 2 x 2 (flip-type x text-orientation) mixed factorial experimental research design. The online consent form system (Qualtrics) block-randomly assigned participants to our between-groups flip-type variable: mirror-reversed versus upside-down text. The PsychoJS software randomized, across trials, our within-participant text-orientation variable: standard versus flipped text.

Four dependent variables tracked the receiver operating characteristics of each participant’s sentence length judgments. These include (1) response precision, (2) response bias, (3) reaction time, and (4) lapses. Conceptually, lapses reflect non-sensory errors. Non-sensory errors can arise from various sources, including inattention, motivation failures, or motor errors. Operationally, we defined lapses as incorrect responses on the shortest (10- and 11-word) and longest (23 and 24-word) sentences—our most extreme stimuli.

To promote reproducibility and generalizability the research design included, respectively, a concurrent direct replication attempt and a concurrent conceptual replication attempt. This resulted in a total of four groups. Two of the four groups judged the length of standard and upside-down sentences. The other two groups judged the length of standard and mirror-reversed sentences. These two pairs of groups provided a conceptual replication attempt because upside-down and mirror-reversed text represent different operationalizations of the flipped-text concept. Each pair of groups provided a direct replication attempt, i.e., two independent participant samples drawn simultaneously from the same population and completing identical experiments. Comparable findings across all four groups would suggest reproducibility, and generalizability across operationalizations of the flipped-text concept.

### A priori sample size rationale and stopping rule

An earlier study showing significant inversion effects across varied stimulus categories [67] (Exp 1, p. 304) reported the following inversion-effect statistics: F(1,14) = 9.37, n = 17. We entered those numbers into the formula shown below (from [94]) to estimate an inversion effect size.


$$\omega^{2}=(\text{a}-1)*(\text{F}-1)/(\text{a}-1)*(\text{F}-\text{a})+(\text{a})*(\text{n}).$$

In that formula, “a” reflects the two levels of the prior study’s [67] inversion variable: upright stimuli versus inverted stimuli. The formula produced the effect size estimate: ⍵^2^ = 0.1975. We then used Table A-6 (p. 538) and equation 8–6 (p. 213) in [94] to estimate sample size. Specifically, we assumed effect size ⍵^2^ = 0.1975, power = 0.9, and *ϕ* = 2.3 given *df* = 1. This generated an estimated sample size of n = 21.49, which we rounded up to 22 participants per group. To minimize P-Hacking [88], we stopped collecting data when each group had 22 participants who met our inclusion criteria.

### Statistical analysis: Psychometric functions

For each of the four groups we constructed two psychometric functions, one for standard text and one for flipped text. The ordinate of the psychometric function reflected the group’s mean proportion of “more-than-17-words” responses. The abscissa comprised the 14 sentence lengths ranging between 10 and 24 words per sentence, excluding the central 17-word length. We used a least-squares procedure to fit the data with the following sigmoidal function.


1/1+exp(−K*(X−Xo))

K and Xo determine the slope and midpoint, respectively, of the sigmoid. In each case, Pearson correlations indicated that the sigmoid significantly fit (p < 6.5^-9) and explained > 94.4% of the response variability. The significant sigmoidal fits permitted estimating the 75% just noticeable difference i.e., the sentence-length threshold. We defined the sentence-length threshold as half the change in sentence length required to alter the “more-than-17-words” response rate from 0.25 to 0.75. Lower thresholds indicate better sentence-length sensitivity i.e., finer sentence-length precision.

### Statistical analysis: Signal detection theory

Using Signal Detection Theory (SDT) [95], we operationally defined “hits” and “false alarms” respectively as “more-than-17-words” responses to sentences containing more or fewer than 17 words. SDT’s d-Prime and beta statistics respectively tracked the precision and bias of each participant’s sentence length judgements, separately for standard text and flipped text.

Computationally, we determined d-Prime using the formula d′ = Z_Hits_ − Z_FalseAlarms_, with the Z-distribution’s SD = 0.5. Accordingly, d-Prime = 0.67 corresponds to non-biased 75% correct performance. We determined beta using the likelihood ratio *β* = Probability Density_Hits_ / Probability Density_False Alarms_. Accordingly, *β* = 1 corresponds to non-biased responding, i.e., using the “More-than-17-word” and “Fewer-than-17-word” response options equally often. A bias toward underestimating sentence length corresponds to *β* > 1. A bias toward overestimating sentence length corresponds to *β* < 1.

Because z-transformations for our SDT statistics required proportions greater than zero and less than one, we adopted the following procedure from [96]. For participants achieving 0 / 35 false alarms, we assumed 0.5 / 35 false alarms. Conversely, for participants achieving 35 / 35 hits, we assumed 34.5 / 35 hits.

### Statistical analysis: Monte Carlo simulations

To avoid the Gaussian-distribution assumption required by parametric tests, we assessed statistical significance non-parametrically. Specifically, we used a Monte Carlo bootstrapping procedure to evaluate median differences among conditions at the 0.05 alpha level. The bootstrapping procedure involved computing a simulated median difference after randomly shuffling the empirically observed data between the experimental conditions under comparison. Repeating this 10,000 times generated a distribution of simulated differences. Statistical significance occurred when the empirically observed median difference exceeded the 95th percentile of the simulated distribution. Larger median differences reflect larger effect sizes. This procedure parallels that used by [97] and the Open Science Framework contains further computational details [https://osf.io/3k5cn].

### Inclusion / exclusion criteria

The statistical analyses included data from participants who satisfied each of two criteria. First, as noted above, the participant had to demonstrate criterion accuracy after at least 20 practice trials (binomial probability p < 0.001). Second, on the subsequent 140 trials for analysis the participants had to achieve at least 62.86% correct (binomial probability p < 0.001).

Each of the 88 participants who met criterion accuracy on practice trials also met criterion accuracy on trials for analysis. We included the data from each of those 88 participants. Nominally, this would suggest a 100% inclusion rate. However, we have no information regarding how many online participants may have started practice trials but subsequently withdrew or failed to reach criterion accuracy.

## Results

### Descriptive statistics

**Fig 4:**
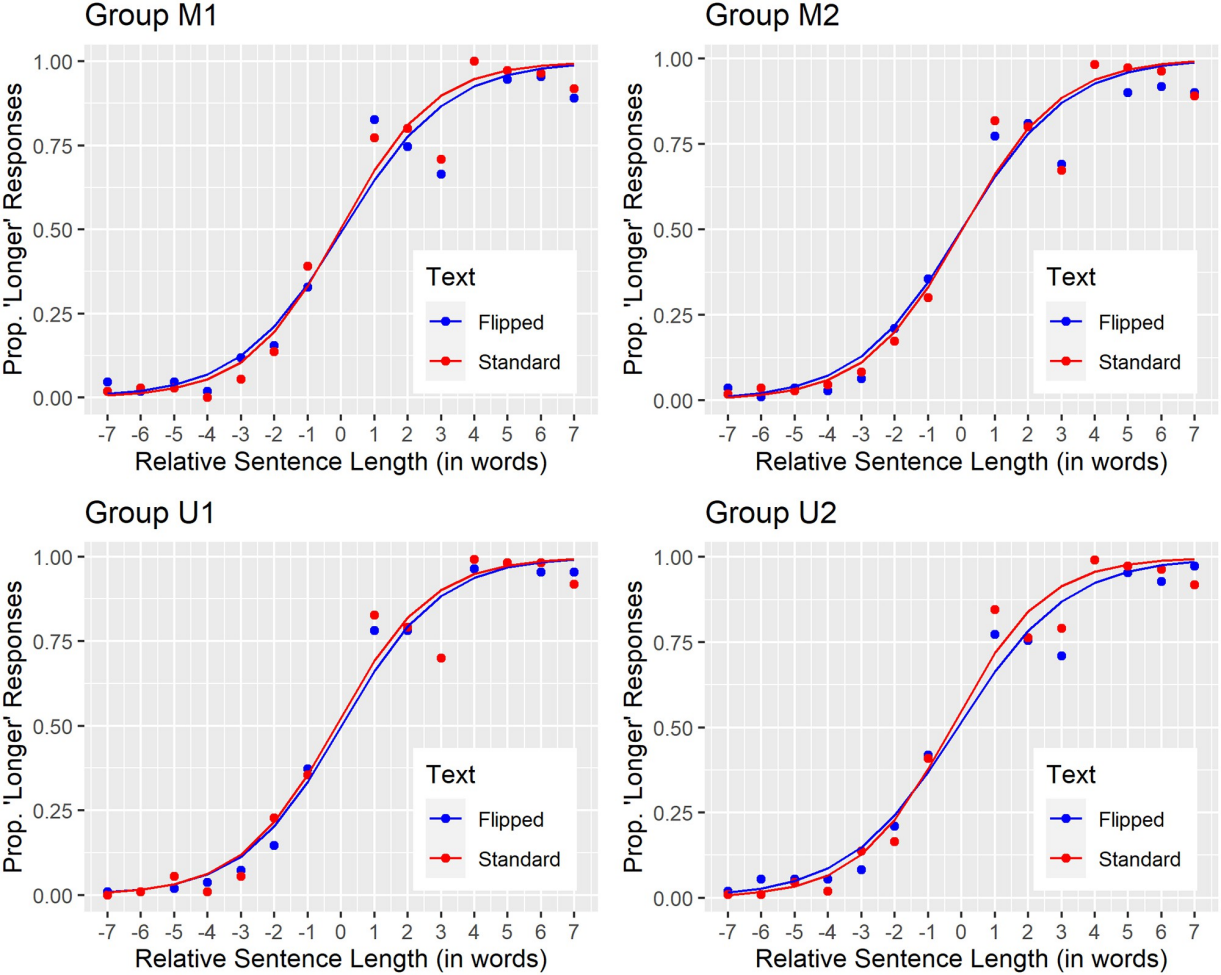
Psychometric functions. Each panel corresponds to a different group of 22 participants. At each relative sentence length, individual data points reflect the mean proportion of “longer” sentence-length responses separately for standard (red) and flipped (blue) text. Standard text (red) generated psychometric functions with only marginally steeper slopes than did flipped text (blue) across groups. This consistent but small “inversion effect” for the precision of sentence-length judgments held for mirror-reversed (Groups M1 and M2) and upside-down text (Groups U1 and U2) alike. The midpoint (point of subjective equality, PSE) of each psychometric function tended toward zero, indicating minimal response bias near the center of the sentence-length range.

Our pre-registered data analysis plan required describing the data with psychometric functions. Fig 4’s psychometric functions reveal similar findings across all four groups. For each group, the best-fitting psychometric functions ranged between the floor and the ceiling as sentence length increased. Also, for each group, standard text (red) and flipped text (blue) generated psychometric functions with similar midpoints and similar slopes. The similar slopes indicate comparable precision when judging the length of standard versus flipped sentences. This contradicts what one would expect given the well-known and large inversion effects in face perception [63, 65, 66], body-position recognition [67], and reading [77]. That said, careful inspection reveals a small yet consistent inversion effect. Specifically, standard text generated slightly steeper psychometric functions than did mirror-reversed text (Groups M1 and M2) or upside-down text (Groups U1 and U2).

We used the psychometric functions in Fig 4 to derive the group summary statistics in Table 1. For standard text, group-mean Just Noticeable Difference (JND) thresholds for sentence length judgments ranged between 1.53 and 1.60 words. Flipping the text impaired the precision of sentence length judgments (elevated JND thresholds) only slightly. Specifically, group-mean JND thresholds for flipped text ranged between 1.61 and 1.81 words. Dividing those group-mean JND thresholds by the mean sentence length of 17 words yielded group-mean Weber fractions. These ranged between 8.98% and 9.41% for standard text. Flipping the text elevated (worsened) the group-mean Weber fractions slightly to between 9.49% and 10.65%. Lastly, across groups and text conditions, the point of subjective equality (PSE) never departed from zero (neutrality) by more than ±0.4 words. This indicates relatively non-biased responding to sentence lengths near the length boundary.

**Table 1:** Descriptive statistics from psychometric functions: Compared to standard text, flipped text generated similar, though marginally worse (higher), sentence-length JND thresholds and Weber fractions. This pattern held for mirror-reversed text (Groups M1 and M2) and upside-down text (Groups U1 and U2) alike. Across conditions, the point of subjective equality (PSE) consistently fell within 0.4 words of non-biased responding (PSE = 0).

	Group M1	Group M2	Group U1	Group U2
Standard Text JND Threshold (Δ Words)	1.53	1.60	1.57	1.53
Flipped Text JND Threshold (Δ Words)	1.72	1.73	1.61	1.81
Standard Text Weber Fraction	8.98%	9.41%	9.22%	9.00%
Flipped Text Weber Fraction	10.10%	10.15%	9.49%	10.65%
Standard PSE (Words)	-.03	.01	-.16	-.31
Flipped PSE (Words)	.05	0	.01	-0.12

### Inferential statistics

#### Precision

**Fig 5:**
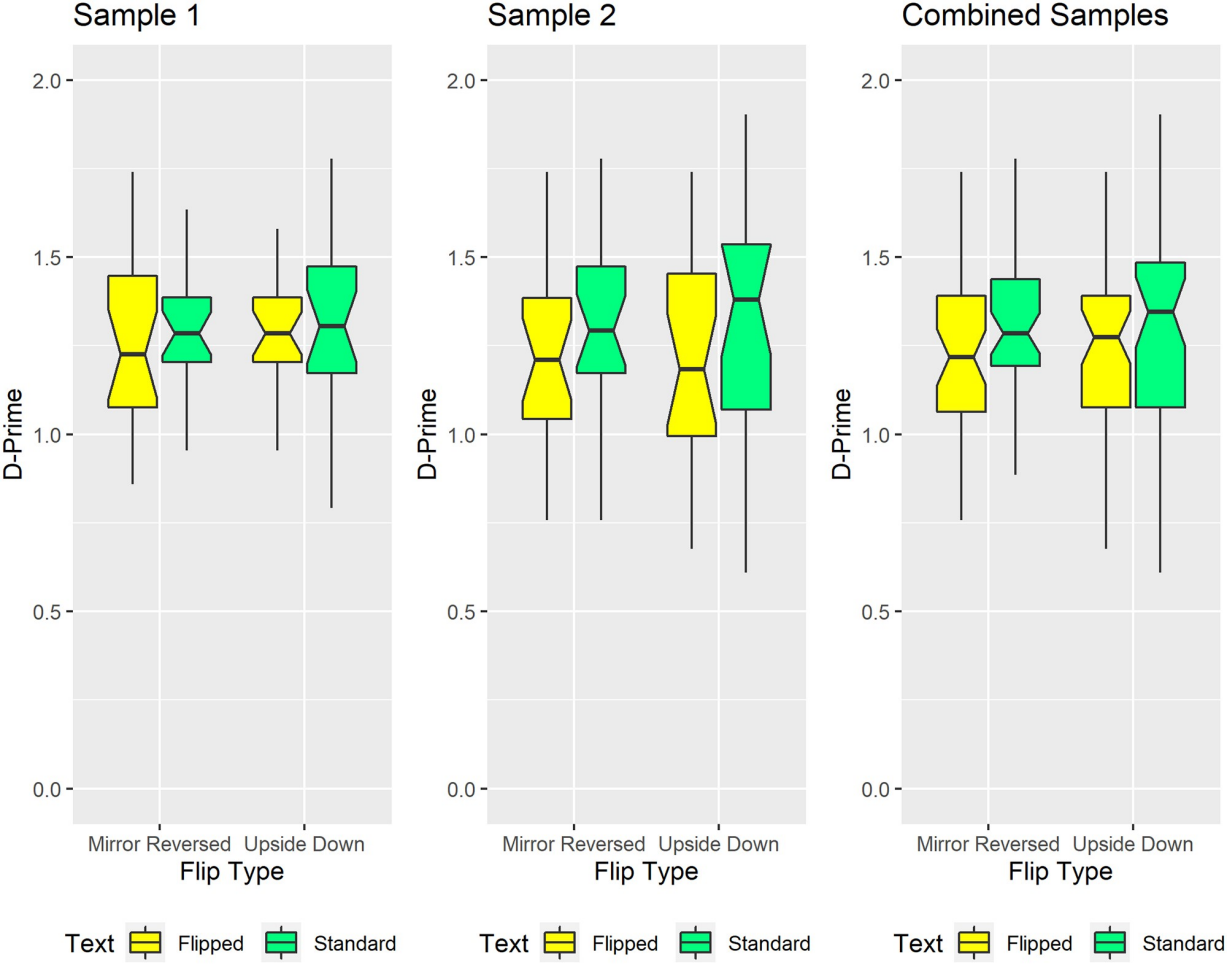
The precision of sentence length judgments. Among the 44 participants in Sample 1, 22 judged mirror-reversed and standard text, and 22 judged upside-down and standard text. Sample 2 (n = 44) was a direct methodological replication of Sample 1. In each sample, flipped text (yellow boxes) slightly impaired the precision of sentence-length judgments relative to standard text (green boxes). The combined samples revealed a statistically significant albeit small inversion effect for sentence length judgments. The upper and lower edges of each colored box respectively reflect the 75th and 25th percentiles, and the central black horizontal line marks the median. The notches within each box extend away from the median by 1.58 * Interquartile Range / sqrt(n), and approximate 95% confidence intervals for comparing medians (98, 99). Whiskers extend to the most extreme empirically observed value no further than ±1.5 * interquartile range from the 75th and 25th percentiles.

The boxplots in Fig 5 show d-Prime, a Signal Detection Theory index of the *precision* with which participants judged sentence length. Higher d-Prime values reflect greater precision. Visually inspecting each sample reveals a slight inversion effect, i.e., slightly lower precision for flipped text (yellow boxes) than for standard text (green boxes). To evaluate this inversion effect statistically, we ran the pre-registered Monte Carlo simulations on the main effect of text-orientation: flipped versus standard text. The preregistered simulations indicated that the inversion effect reached statistical significance in Sample 2 (p = 0.021) but not in Sample 1 (p = 0.1261). An exploratory simulation combined the data from the two samples (n = 88) and revealed a statistically significant (p = 0.0067) but small inversion effect. Specifically, relative to standard text, flipped text impaired precision by 0.0976 d-Prime units. For context, this effect size corresponds to non-biased responding at 90.7% correct for standard text compared to 89.0% correct for flipped text. The main effect of flip-type (mirror-reversed versus upside-down text) was non-significant.

#### Reaction time

**Fig 6:**
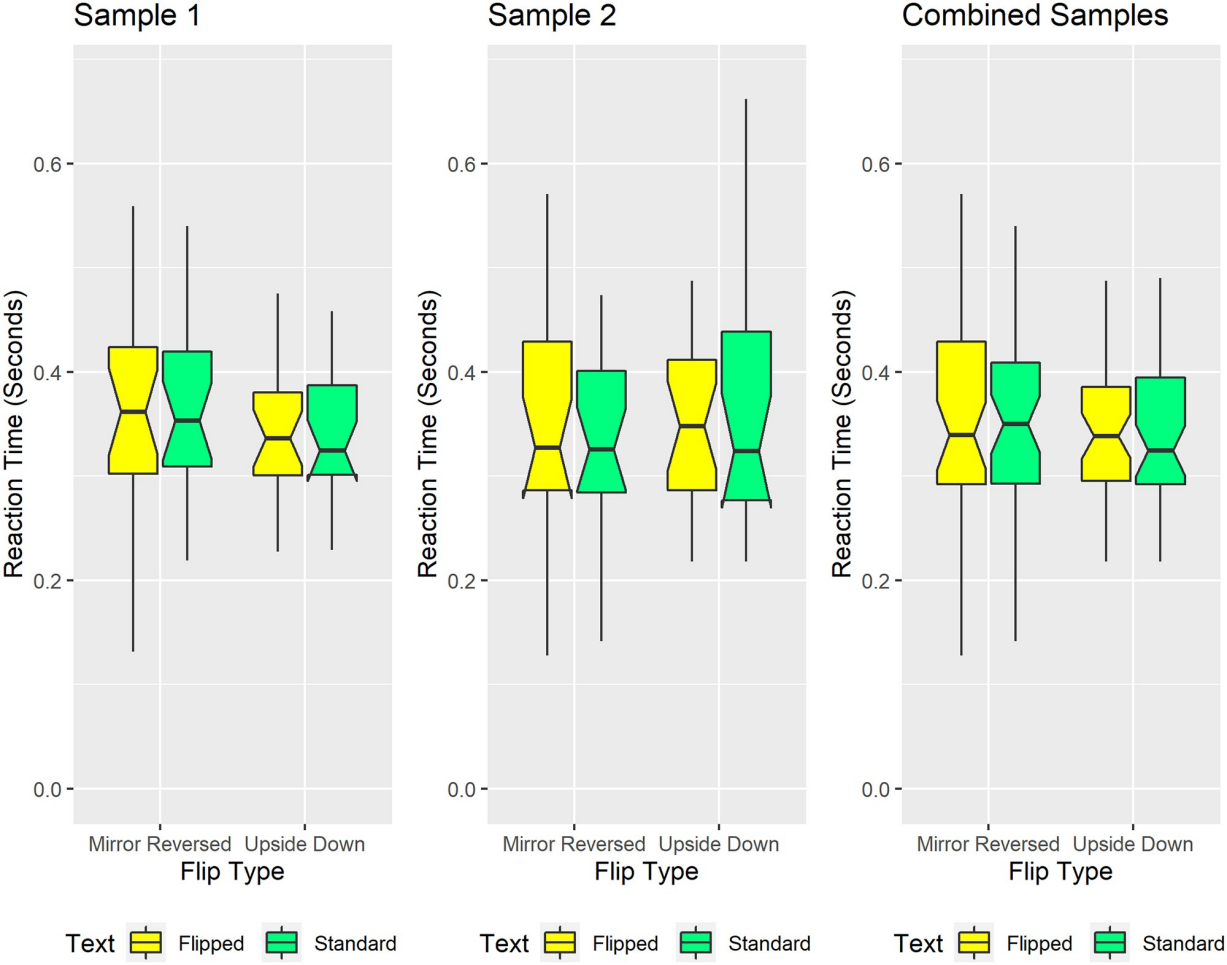
Reaction Times for sentence length judgments. Participants responded with comparable speed across conditions. Conventions remain the same as in Fig 5. Some of the colored boxes show downward-pointing protrusions. These reflect distributions skewed such that the 25th percentile falls within the median’s 95% confidence interval, i.e., within the box’s notched region [98, 99].

The boxplots in Fig 6 show reaction times for sentence length judgements. Visual inspection reveals comparable reaction times across conditions and groups. Regarding effect size, only 38 msec separated the fastest (sample 2, standard text) and slowest (sample 1, mirror-reversed text) median reaction times. Correspondingly, pre-registered Monte Carlo simulations indicated non-significant main effects and interaction effects within each sample. Exploratory Monte Carlo simulations that combined the samples also indicated non-significant main and interaction effects. These null findings argue against speed-tradeoffs causing the small -albeit statistically significant- inversion effect in response precision (see Fig 5).

#### Response bias

**Fig 7:**
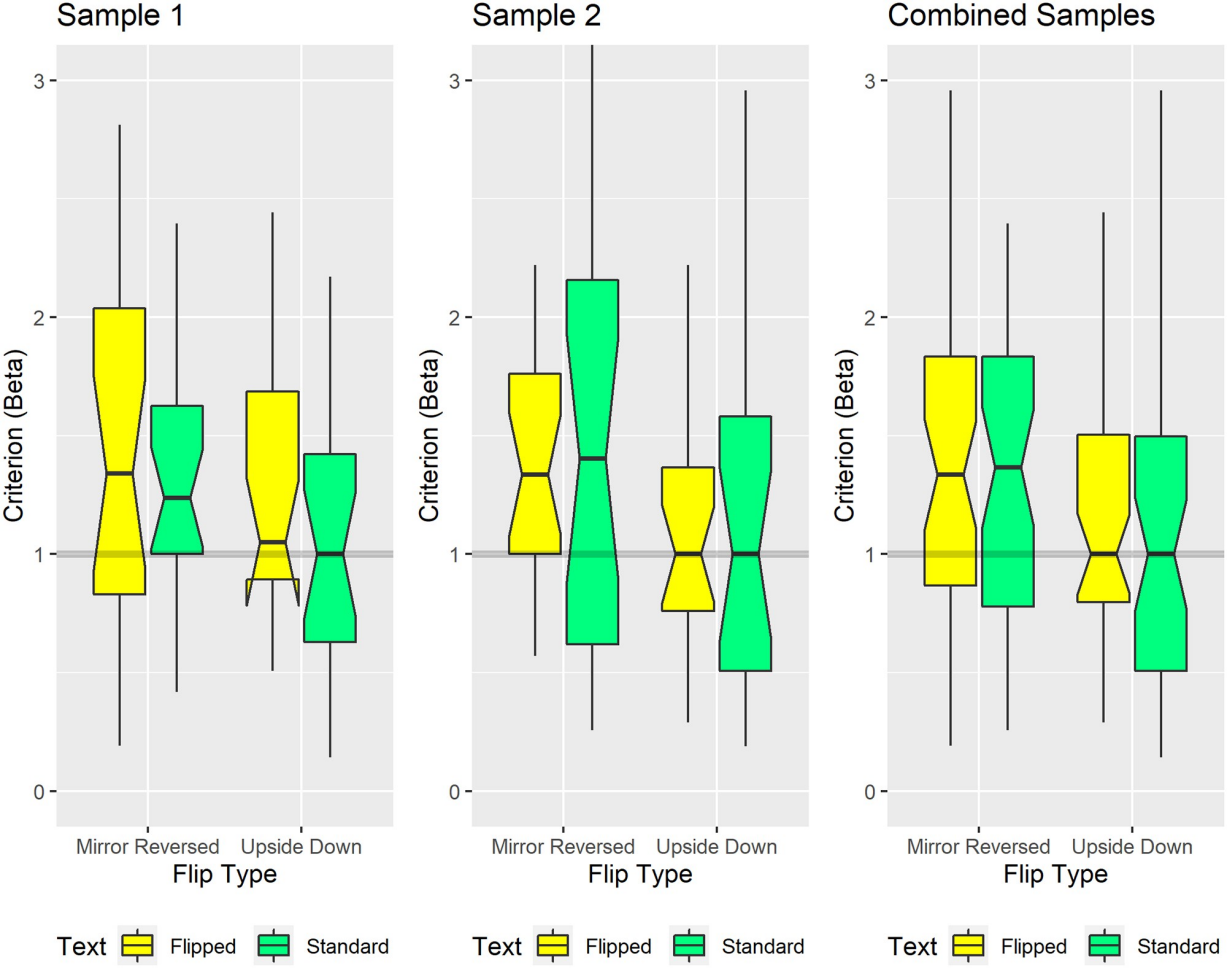
Biases in sentence length judgments. The gray horizontal line at 1 marks unbiased responding, i.e., equal usage of the “more-than-17-word” and “fewer-than-17-word” response options. The mirror-reversed groups exhibited a bias toward underestimating sentence length, shown by median criterion (Beta) values greater than 1. The upside-down groups judged sentence length in a relatively unbiased manner, shown by median criterion (Beta) values near or at 1. Conventions remain the same as in Fig 5. In Sample 1, the yellow box for the upside-down group’s flipped condition shows downward-pointing protrusions. This reflects a distribution skewed such that the 25th percentile falls within the median’s 95% confidence interval, i.e., within the box’s notched region [98, 99].

The boxplots in Fig 7 show the criterion (Beta), a Signal Detection Theory index of the *bias* with which participants judged sentence length. Within each plot the gray horizontal line at 1 marks neutral responding, i.e., using the “More-than-17-word” and “Fewer-than-17-word” response options equally often. A bias toward underestimating sentence length corresponds to criterion (Beta) values greater than 1. A bias toward overestimating sentence length corresponds to criterion (Beta) values less than 1.

Surprisingly, visually inspecting Fig 7 reveals that response biases varied systematically between groups, rather than within groups. Specifically, participants randomly assigned to our mirror-reversed groups tended to underestimate the length of mirror-reversed sentences (yellow boxes) *and* standard sentences (green boxes). By contrast, participants randomly assigned to our upside-down groups tended to neutrally judge the length of upside-down sentences (yellow boxes) *and* standard sentences (green boxes). Stated differently, the main effect of flip-type (mirror-reversed versus upside-down) mattered more than did the main effect of text-orientation (flipped versus standard).

Monte Carlo simulations support these visually evident patterns. First, our pre-registered Monte Carlo simulations showed a non-significant main effect of text-orientation (flipped versus standard) within each sample. This effect remained non-significant even after increasing the statistical power by combining the samples in exploratory simulations. Second, exploratory simulations on the combined samples showed that our mirror-reversed groups underestimated sentence length significantly more than did our upside-down groups (p = 0.0023).

Regarding effect size, the mirror-reversed groups’ median Beta value (1.351) exceeded that of upside-down groups (1.0; perfect neutrality) by 35.1%. Equivalently, one can model the mirror-reversed groups’ *underestimation* bias by altering the miss *and* false alarm rates relative to those of the upside-down groups’ unbiased responses. An example entails increasing the miss rate from 9.6% to 21.6% *and* reducing the false alarm rate from 9.6% to 4.1%. Indeed, these miss and false alarm rates generate the empirically observed median criterion (Beta) *and* median d-Prime values from the mirror-reversed and upside-down groups. Misses reflect sentence length underestimates; false alarms reflect sentence length overestimates.

#### Lapses

Our preregistered methods operationally defined lapses as incorrect responses on the two longest and two shortest sentence lengths. These relatively extreme sentence lengths correspond to more than three times the subsequently observed median JND threshold in each condition.

**Fig 8:**
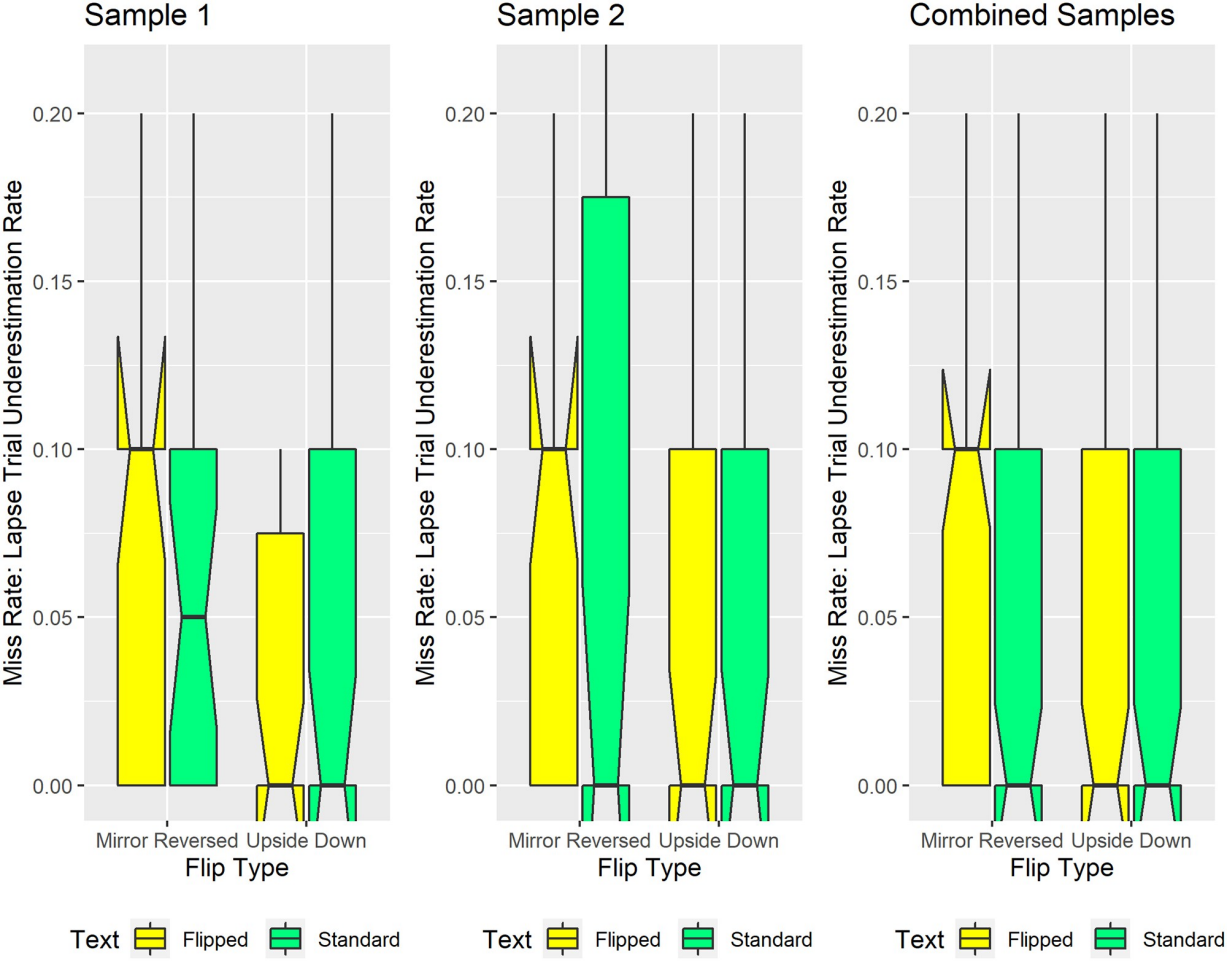
Lapses that reflect sentence length underestimates. The ordinate reflects the proportion of trials when participants underestimated sentence length, incorrectly classifying 23-word or 24-word sentences as having “Fewer-Than-17-Words”. Mirror-reversed text consistently generated more sentence-length underestimates, on median, than did the other conditions. The mirror-reversed text also produced distributions skewed such that the 75th percentile equaled the median. The corresponding box plots show upward-pointing protrusions. Conversely, other experimental conditions produced distributions skewed such that the 25th percentile equaled the median. Those conditions show downward-pointing protrusions. Conventions remain the same as in Fig 5.

Fig 8 tracks lapses that correspond to Signal Detection Theory “misses”. These occurred when participants underestimated sentence length by responding “Fewer-Than-17-Words” to sentences containing 23 or 24 words. Visual inspection reveals that mirror-reversed text consistently generated the highest median rate of sentence-length underestimates; 10% of the 23-word and 24-word sentence trials. Notably, for the combined samples (Fig 8, rightmost panel), all experimental conditions except the mirror-reversed condition generated 0% underestimation rates, on median. This ten percentage-point difference in median underestimation rates reflects the effect size for mirror reversing the text. Exploratory Monte Carlo simulations on the combined samples (Fig 8, right panel) confirmed this significant flip-type-by-text-orientation interaction (p = 0.0024). Specifically, on median, the mirror-reversed condition generated significantly more sentence-length underestimates than did each of the other conditions (p<0.001) (Fig 8, right panel).

**Fig 9:**
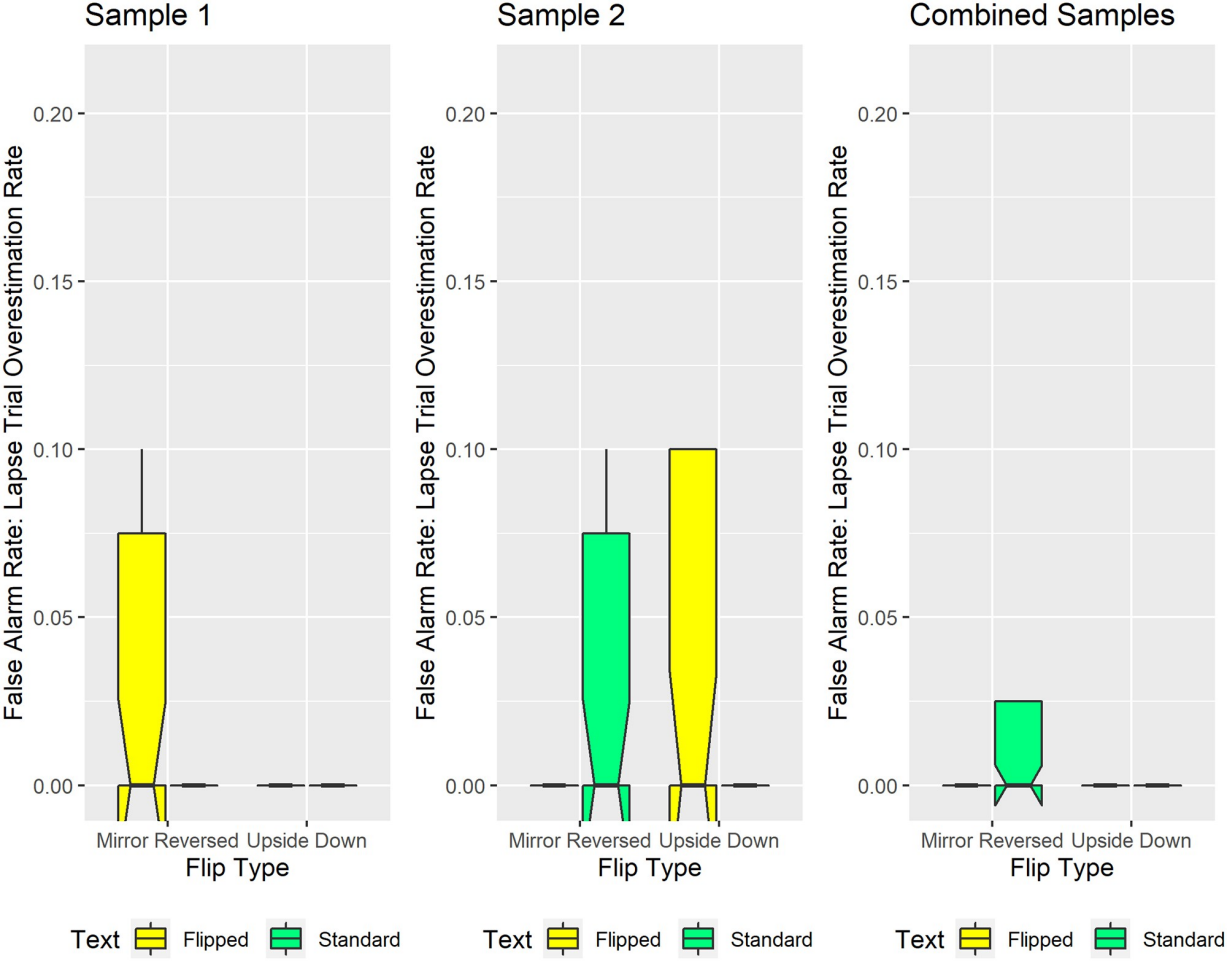
Lapses that reflect sentence length overestimates. The ordinate reflects the proportion of trials when participants overestimated sentence length, incorrectly classifying 10-word or 11-word sentences as having “More-Than-17-Words”. Median overestimation rates remained identical and low (0% of trials) across experimental conditions. Some conditions produced distributions skewed such that the 25th percentile equaled the median. Those conditions show downward-pointing protrusions. Conventions remain the same as in Fig 5.

Further evidence for the specificity of this under-estimation effect comes from contrasting Fig 8 with Fig 9. Fig 9 tracks lapses that correspond to Signal Detection Theory “false alarms”. These occurred when participants overestimated sentence length by responding “More-Than-17-Words” to sentences containing 10 or 11 words. Visually inspecting Fig 9 reveals that, on median, each experimental condition generated sentence length overestimates on 0% of trials containing 10 or 11 words. Given that the median overestimation rate remained identical across conditions (effect size = 0), we did not conduct statistical analyses on Fig 9‘s data.

In summary, the Lapse analyses demonstrate that participants significantly underestimated the length of mirror-reversed—but not upside-down, nor standard—sentences. In the Discussion we address how the specificity of this inversion effect relates to scene syntax [78–80, 85].

#### Sentence length heuristic and the mischievous sentence

Recall that during our study’s demonstration and practice phases, we primed participants with a sentence-length heuristic: 17-word sentences typically span ~1.5 text lines. Per our pre-registered hypotheses and research design, we probed participants’ use of this heuristic via our “mischievous sentence”. The mischievous sentence contained only 16 words, yet appeared in three consecutive lines of text. Specifically, it began near an edge of its first line, spanned its second line, then ended near the opposite edge of its third line. This differed from the other 16-word sentences, which each spanned no more than two text lines. If used, our heuristic would generate more errors (sentence length *overestimates*) on the 3-line-16-word mischievous sentence than on the 2-line-16-word sentences.

**Fig 10:**
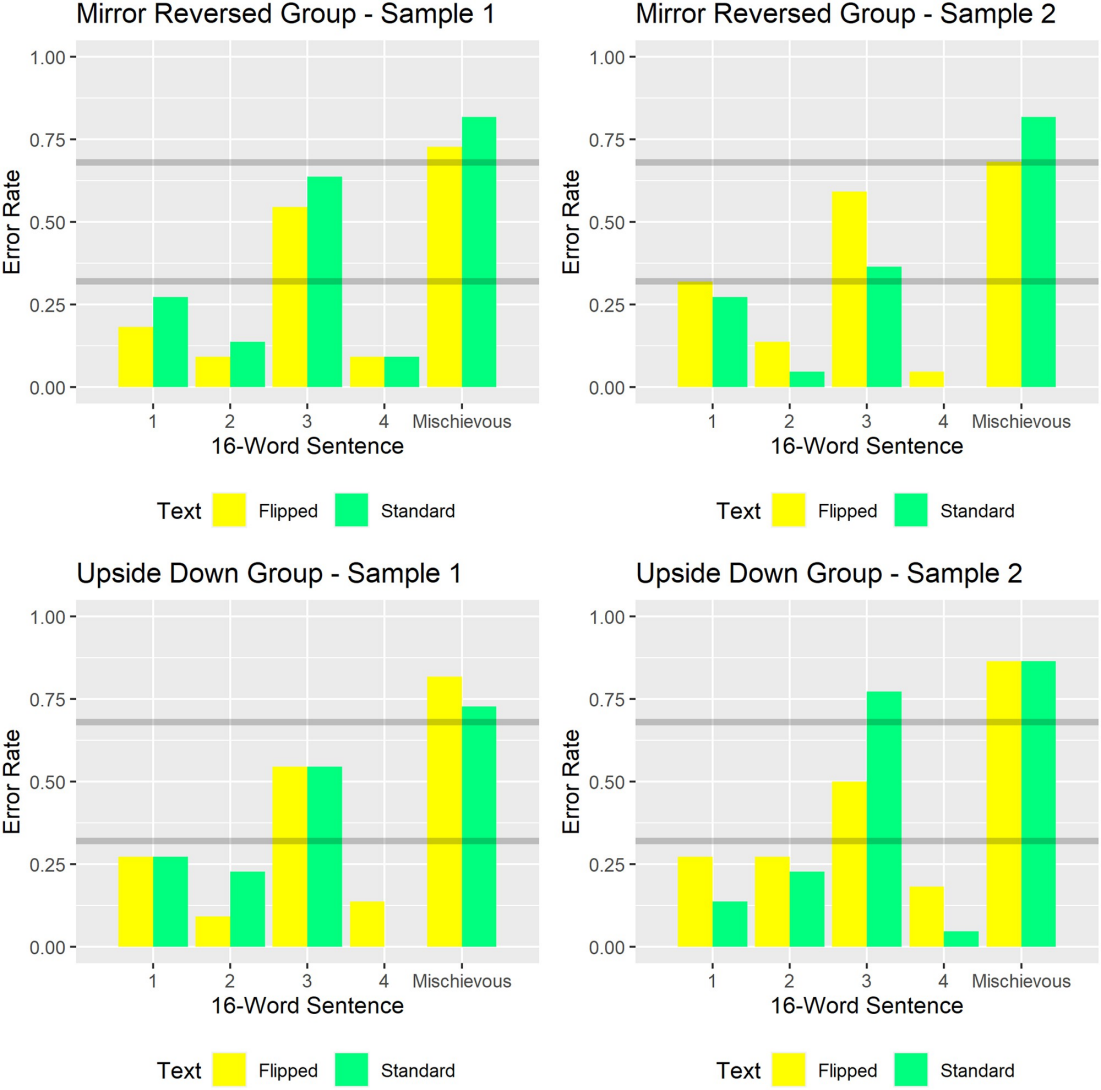
Sentence length heuristic and the mischievous sentence. The four panels correspond to the four groups of 22 participants. Each ordinate reflects how often participants overestimated sentence length; incorrectly judging 16-word sentences as having more than 17 words. Gray horizontal lines at 0.32 and 0.62 respectively reflect error rates significantly (p<0.05) better and worse than pure guessing. In each group, the 3-line, 16-word “mischievous” sentence generated significantly (p<0.05) worse-than-chance performance on flipped (yellow) and standard (green) text alike. This contrasts with consistently lower error rates for sentences 1–4, which each also contained 16-words but spanned two rather than three text lines. The specificity and reproducibility of the mischievous sentence effect suggest that participants judged sentence length by heuristically counting lines, not by explicitly counting words.

Fig 10 compares error rates on the 16-word sentences, separately for each group. The gray horizontal lines at 0.32 and 0.68 respectively reflect error rates better and worse than random responding (binomial probability < 0.05). Visual inspection reveals that each group made more errors on the 16-word mischievous sentence than on the other four 16-word sentences. Moreover, the mischievous sentence generated error rates significantly *worse* (higher) than predicted by mere random responding (upper gray line at error rate = 0.68). This significant mischievous sentence effect replicated across all eight experimental conditions: flipped (yellow bars) and standard (green bars) text in each of the four groups. By contrast, the other 16-word sentences typically generated error rates lower than expected by chance (lower gray line at error rate = 0.32). The one exception (sentence 3) generated worse-than-chance (higher) error rates in one experimental condition, and chance-level error rates in the remaining seven experimental conditions. Overall, the specificity in Fig 10‘s error patterns suggest that participants judged sentence length by heuristically counting text lines, not by explicitly counting words.

Lastly, our pre-registered data analyses for the mischievous sentence required conducting Monte Carlo simulations to test the sentence-by-text-orientation interaction effects. Each of those simulations showed non-significant interactions. Likewise, exploratory simulations on mischievous sentence trials showed non-significant interactions between flip-type (mirror-reversed versus upside-down) and text-orientation (standard versus flipped). To summarize, the findings from our mischievous sentence manipulation suggest that, regardless of flip-type and text-orientation, participants judged sentence length by heuristically counting text lines.

## Discussion

Short sentences play a critical role in readability [10]. Short sentences also promote social justice through accessibility and inclusiveness. Despite this, much remains unknown about sentence length perception—an important factor in producing readable writing. Accordingly, we conducted the present psychophysical study to address the applied-research question of how precisely people perceive sentence length. We also sought to link sentence length perception to prior basic research on fundamental visual phenomena. These basic visual phenomena include numerosity sensitivity, perceptual learning, and scene syntax. Participants viewed real-world full-page text samples and judged whether a bolded target sentence contained more or fewer than 17 words. The experiment yielded four main findings, which we consider in turn.

First, naïve participants precisely and quickly perceived sentence length in real-world text samples. Regarding precision, participants achieved ~90% correct responding on median, with median sentence-length Weber fractions ranging between 8.98% and 10.65%. Regarding speed, median reaction times ranged between 300 and 400 milliseconds. Moreover, 88 of 88 naive participants met the inclusion criteria. Taken together, these findings demonstrate the ease with which our naive adult participants perceived the length of target sentences in real-world English text samples.

Second, flipping the text generated no reaction-time cost and nearly no loss in the precision of sentence length perception. The text-orientation effect size corresponded to non-biased 90.7% correct responding for standard text compared to non-biased 89.0% correct responding for flipped text. This robustness to global text orientation variability contrasts sharply with the large inversion effects previously reported for diverse stimuli and tasks. These include the perception of faces [62–66], body parts [67], mammograms [68], artificial objects (“greebles”) [69, 70], oriented shapes [71], change detection [72, 73], lexical decisions [74, 75], word identification [76], and reading [77]. The nearly orientationally invariant sentence length perception observed here aligns well with predictions from the numerosity sensitivity hypothesis. The numerosity sensitivity hypothesis parsimoniously posits that sentence length perception depends only on mechanisms already used to quantify other stimuli in the environment. Prior behavioral [42–46] and physiological [36, 47–60] research has shown that numerosity-sensing mechanisms do not depend on specific stimulus features, which would include global text orientation.

Third, our three-line 16-word “mischievous sentence” consistently generated more errors—specifically, sentence length overestimates—than did any of our two-line 16-word sentences. Also, unlike any of our two-line 16-word sentences, our three-line 16-word “mischievous sentence” consistently generated more errors (sentence-length overestimates) than predicted by mere random responding. The reproducibility and specificity of this finding suggests that participants took advantage of the heuristic that 17-word sentences typically span ~1.5 text lines. This in turn implies that the participants’ high speed, high precision, and largely orientationally invariant sentence-length judgments reflect subitizing text lines [31–33], not explicitly counting words. Relatedly, one might interpret this finding as a novel instance of “groupitizing” [33, 41]—perceptually grouping a sentence’s spatially proximal words into subitizable text lines. In any case, the speed, precision, and general orientational invariance of participants’ sentence-length judgments align well with the subitizing [31–33] specified by our numerosity sensitivity hypothesis.

Fourth, participants significantly underestimated the length of mirror-reversed sentences—but not upside-down, nor standard sentences. Evidence for this came from our lapse analysis. Here, participants exhibited a significant bias toward classifying 23- and 24-word sentences as having fewer than 17 words, but only for mirror-reversed text. The specificity in underestimating mirror-reversed sentence length partially matches predictions from our scene syntax hypothesis. In preregistration, we predicted that participants would underestimate flipped-sentence length because mirror-reversing the text or flipping it upside-down repositions words from high-probability to low-probability locations. The data support the predicted underestimation-bias for mirror-reversed text only.

Given that mirror-reversed text and upside-down text each occur rarely in real world settings, why would significant sentence-length-underestimates occur only for mirror-reversed text? One possible explanation comes from research demonstrating that spatial anchors influence visual search [80, 100, 101]. Anchors predict the likely position of other stimuli in real-world scenes. For example, the nose serves as a spatial anchor in face perception [102–107]. In the present study, left-justified text may have served as a spatial anchor. Our standard and upside-down sentences had the typical real-world left-justified right-ragged English text orientation, and generated no biases in sentence length perception. By contrast, our mirror-reversed sentences had a highly atypical right-justified left-ragged English text orientation, and generated significant sentence-length underestimates. Earlier research has shown that the English language’s left-to-right reading direction creates left-side prioritization biases in letter encoding [108] and perceptual spans during eye movements [109]. It therefore seems possible that our participants’ extensive practice with the English language’s left-to-right reading direction created visual search priority maps anchored to left-justified text. Mirror-reversing the text would reposition the sentence’s lateral-justification from high-priority-left to low-priority-right. The resulting spatial mismatches may have generated “misses” and the corresponding significant sentence length underestimates that occurred uniquely for mirror-reversed text. If so, our finding that participants significantly underestimated sentence length only for mirror-reversed text suggests novel evidence for left-lateral anchoring in scene syntax.

While left-laterally anchored scene syntax would account for the significant sentence-length underestimates observed here, we emphasize that our pre-registered hypotheses did not include that explanation. In fact, left-lateral anchoring occurred to us only after the data showed significantly greater sentence-length underestimates for mirror-reversed text than for standard and upside-down text. The post hoc nature of this explanation warrants future attempts to replicate the significant sentence-length underestimation bias observed here for mirror-reversed text.

Other future studies might provide new insights about sentence length perception by building on the present experiment’s task and stimuli. Our stimuli comprised real-world text examples containing a bolded target sentence among non-bolded distractor sentences. Our two-step task required (1) searching for the bolded target sentence and then (2) judging its length relative to a reference length. However, real-world text pages often contain no bolded sentences, and their absence would complicate the visual search component of the task. This suggests a future conventional visual search experiment comprising non-bolded short distractor sentences and, on half the trials, a non-bolded target sentence of reference length. Participants would report “target-absent” or “target-present” on each trial. Here, sentence length—rather than bold font—would distinguish targets from distractors, paralleling real-world text conditions. A finding that performance on this visual search task benefits from a line-counting heuristic—as our results suggest—could help writers produce more readable writing.

## Conclusion

Short sentences improve readability [10]. Readability matters for broad audiences. To reach broad audiences writers need sensitivity to sentence length, yet much remains unknown about sentence length perception in writers—indeed, any adults. Here, we used real-world English text samples and psychophysical methods to investigate sentence length perception in naive adults. We manipulated sentence length by varying the number of words per sentence -a metric that commonly determines text readability and grade level. Regarding basic vision science, we found that sentence length perception remained nearly unchanged after flipping real-world text samples upside-down. This differs from the large inversion effects that characterize many highly practiced, real-world perceptual tasks involving canonically oriented stimuli, most notably face perception and reading. Additionally, our finding that participants significantly underestimated sentence length only for mirror-reversed text suggests a novel demonstration of visual spatial anchoring. Our results also have implications for writing instruction and pedagogy. Most notably, we found that naive adults quickly and precisely perceived sentence length in real-world text samples. Their error patterns demonstrated that they accomplished this high speed and precision by heuristically counting text lines, not by explicitly counting words. This suggests practical advice that writing instructors might offer students. When copy editing, students can quickly identify their long sentences via a line-counting heuristic, e.g., “a 17-word sentence spans about 1.5 text lines”. Students can subsequently improve a long sentence’s readability and inclusiveness by following a simple rule. Omit needless words.

## Supporting information

S1 Fig Standard two-line 17-word sentence. (JPG)

S2 Fig Mirror-reversed two-line 17-word sentence. (JPG)

S3 Fig Upside-down two-line 17-word sentence. (JPG)

S4 Fig Standard three-line 16-word mischievous sentence. (JPG)

S5 Fig Mirror-reversed three-line 16-word mischievous sentence. (JPG)

S6 Fig Upside-down three-line 16-word mischievous sentence. (JPG)
