# Supplementary figures and images for: Omit needless words: Sentence length perception
Source: PLoS One. 2023 Feb 24;18(2):e0282146. doi: 10.1371/journal.pone.0282146 (PMC9955962; doi:10.1371/journal.pone.0282146)

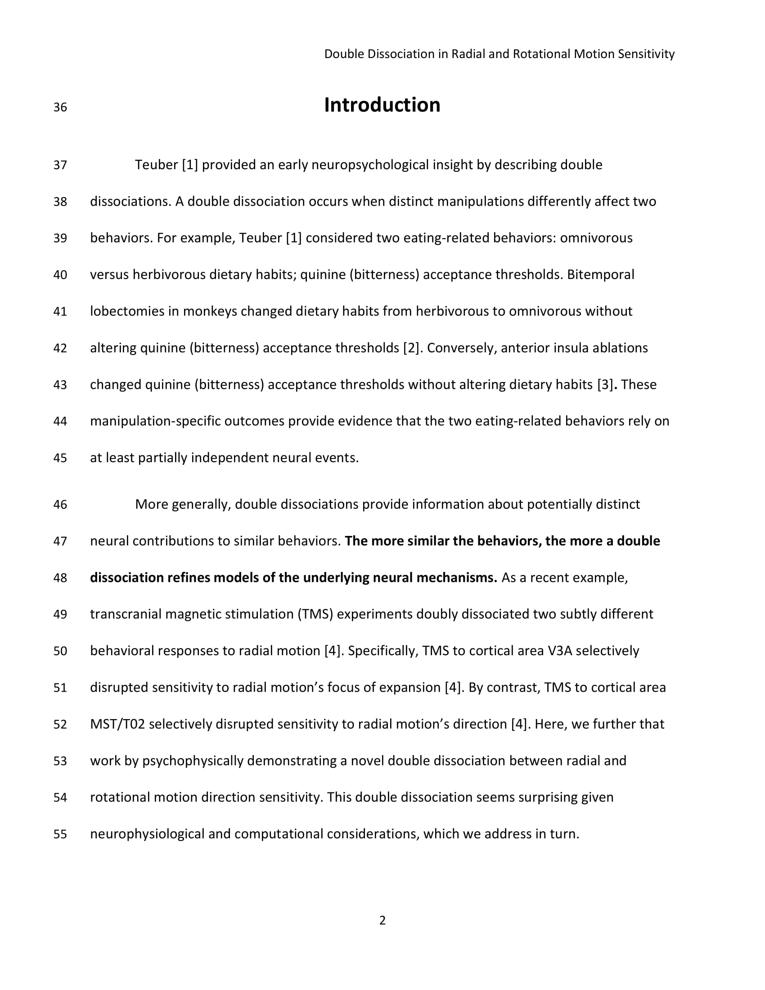

Supplement: S1 Fig — (JPG) [file pone.0282146.s001.jpg]

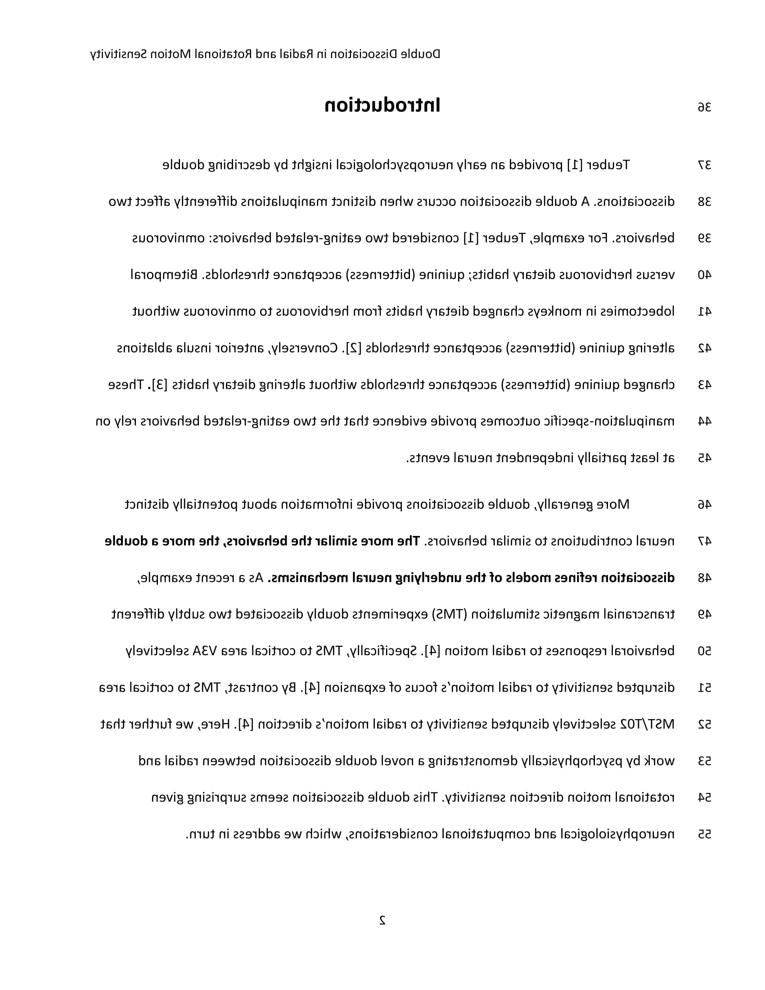

Supplement: S2 Fig — (JPG) [file pone.0282146.s002.jpg]

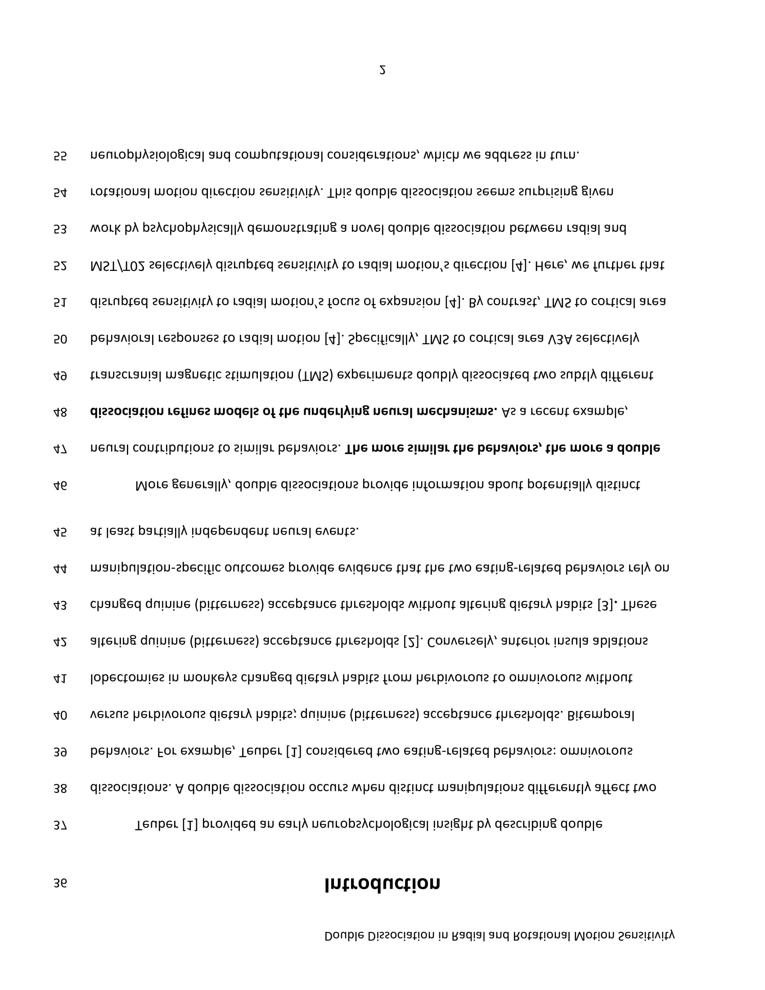

Supplement: S3 Fig — (JPG) [file pone.0282146.s003.jpg]

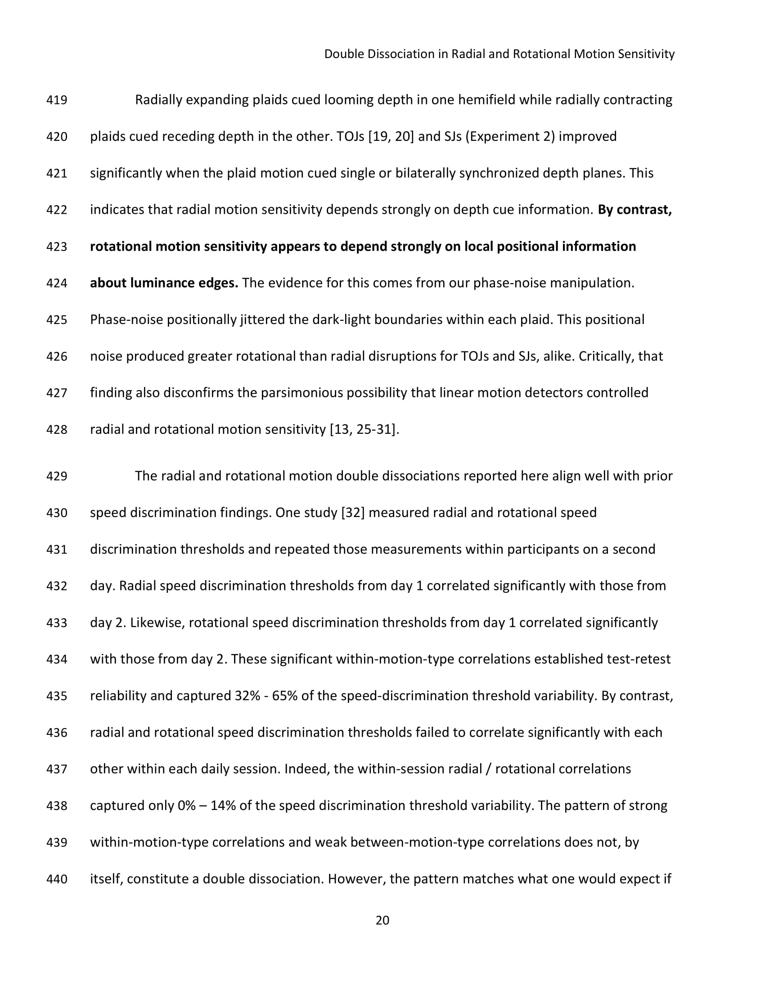

Supplement: S4 Fig — (JPG) [file pone.0282146.s004.jpg]

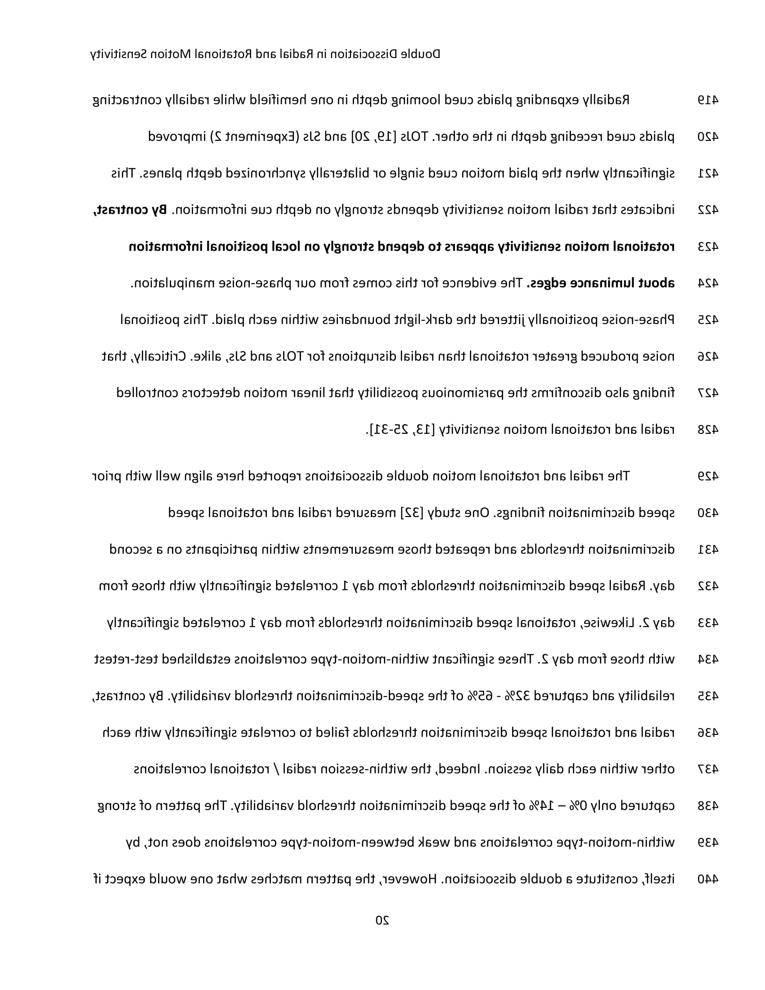

Supplement: S5 Fig — (JPG) [file pone.0282146.s005.jpg]

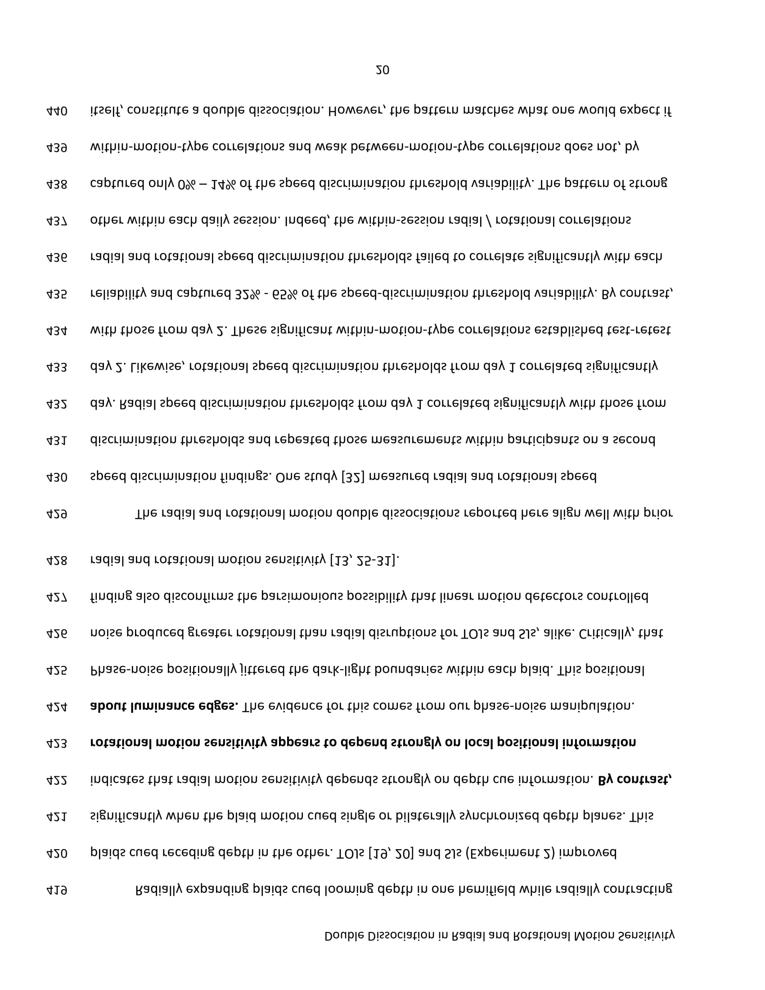

Supplement: S6 Fig — (JPG) [file pone.0282146.s006.jpg]
